# Treatment of Attention Deficit Hyperactivity Disorder (ADHD) in Pregnant Women: A Systematic Review of Cohort Studies

**DOI:** 10.1192/bjo.2022.250

**Published:** 2022-06-20

**Authors:** Indu Surendran, Kalpa Wijesinghe, Joe Johnson

**Affiliations:** 1 St Helens and Knowsley Teaching Hospitals NHS Trust, Prescot, United Kingdom; 2 Merseycare NHS Foundation Trust, Prescot, United Kingdom

## Abstract

**Aims:**

The aim of this systematic review is to identify and appraise pharmacological options available for management of ADHD in pregnant women and adverse effects of ADHD medications on pregnancy and foetus (malformations or long-term effects).

**Methods:**

Systematic review of prospective or retrospective cohort studies, available on this topic till September 2021 after PubMed and MEDLINE search, carried out by 2 reviewers independently. The preliminary search was conducted in March 2021, though the reviewers carried out timely cross-referencing as required. All cohort studied except those with ambiguous methodology were included. The data were further extracted using Microsoft excel after Quality Assessment was completed using NewCastle Ottawa Scale. A narrative synthesis was undertaken as meta-analysis was not feasible owing to heterogeneity between studies included.

**Results:**

Eighteen Cohort studies were included (N = 28227 pregnant women with ADHD) of which 16 were deemed as good quality. Multiple confounders were identified.

The review noted that use of stimulants/non-stimulants reduced symptoms and improved functionality in these expectant mothers. Findings from our review overall indicate low risk of developing malformations with ADHD medications, with only Methylphenidate (notably cardiac) and Modafinil showing slightly increased though statistically significant risk. We also noted slightly increased risk for reduced Apgar scores, abortions on maternal request, pre-eclampsia and preterm births. There was no conclusive association noted between neuro-developmental delay or future ADHD in baby/child.

**Conclusion:**

A case-by-case approach needs to be adopted for every patient, looking at how ADHD affects daily functioning and balancing that against adverse pregnancy outcomes. Also, innovative practices like drug holidays, as required medications, drug free trial while planning pregnancy etc. will help practitioners streamline the treatment of this group of patients better. Issues like research being restricted to certain countries, small sample size, record-based analysis, issues in ascertaining adherence, confounding factors, ethical conundrums etc. were noted.

